# Technology applications in shoulder replacement

**DOI:** 10.1186/s10195-019-0535-1

**Published:** 2019-07-17

**Authors:** G. Porcellini, L. Tarallo, M. Novi, F. Spiezia, F. Catani

**Affiliations:** 1 Department of Orthopaedics and Traumatology, Modena Policlinic, Modena, Italy; 2 Department of Orthopaedics and Traumatology, University Hospital of Pisa, Pisa, Italy

## Abstract

The advancement of technologies in orthopaedic surgery should provide the surgeon with precise and trustworthy support for pre-operative planning, intra-operative guidance and post-operative follow-up. The request for greater accuracy, predictable results and fewer complications, is the engine of digital evolution in pre-operative planning and computer-assisted surgery (CAS). It is an evolution rather than a revolution, and in the last few years these developments have begun to involve shoulder replacement surgery, too.

Key points for ensuring satisfactory long-term results of both total anatomic and reverse shoulder arthroplasty are accuracy in restoring a correct glenoid version and joint line, and achieving implant stability with proper soft tissue tension. Glenoid loosening is among the main causes of implant failure.Variations in morphology of scapula and humerus, due to fractures or osteoarthritis, can lead to severe anatomic changes. Pathological glenoid inclination, such as Habermayer’s type-2 and type-3, as well as a glenoid retroversion greater than 15°, if not corrected, cause uneven stress and higher risk of loosening [1, 2].

The same accuracy should also be applied to the humeral side, but today we continue to follow conventions, such as 20° of retroversion, instead of considering a correct patient-specific functional alignment of the prosthetic components.

The standard 2-dimensional imaging and standard surgical instrumentation are insufficient for preoperative planning and execution, taking into account all these factors. The preoperative phase needs CT-based planning to understand geometries and morphology of the patient; then technology should guide the surgeon to “do what he planned”.

Several technological instrumentations are emerging to support orthopaedic surgeons; however, not all innovations guarantee sufficient accuracy to achieve a better outcome. The patient-specific instrumentation technology (PSI) was initially promising, but has shown some limitations recently. It represents a custom-made guide for the positioning of the glenoid component. It can be obtained by pre-operative 3D-CT images planning. This patient-specific template fits the glenoid and permits the correct positioning of the base plate and screws by using a pre-planned drilling guide [3]. This technique has been inherited from knee, hip and spine surgery, where the benefits are well known, but its real impact in shoulder arthroplasty is not clear. This system has certainly improved pre-operative planning by better classifying concentric and eccentric osteoarthritis, dysplasia and glenoid erosion. Although improving accuracy compared to standard procedures, PSI cannot guarantee complete intra-operative control, because it is a “passive technology” based only on bone morphology without considering cartilage.

## CT-based navigation in shoulder surgery

### Why do we need navigation and computer-assistance in shoulder surgery?

Pre-operative 3D surgical planning and computer navigation technology can increase the accuracy and reproducibility of the implantation of the glenoid component, especially in terms of position and orientation of the glenosphere and screws in reversed arthroplasty [4–6]. By observing particular clinical and radiological indications, CAS reduces early and mid long-term failure for wrong indications or poor surgical techniques. Consequently, a better clinical outcome for patients is expected.

Moreover, the navigation system seems to be a valuable teaching tool for speeding up the surgeon’s learning curve, especially in centres where the number of procedures is limited. Surgery can be accurate and reproducible soon, even if the surgeon is only performing a small number of replacements per year.

However, along with these advantages there are some limitations. The actual navigation systems need larger surgical approaches, for two reasons: relatively cumbersome instrumentation, especially the tracker positioned on the coracoid; and the exposure of the glenoid, the anterior part of the scapular neck and coracoid for probing. Other possible complications are coracoid fracture or neurovascular injuries from the pins used for the array.

With routine use of navigation, other technical issues come out: the base plate orientation is currently not navigated as well as the position of the screws. Navigated jigs and screwdrivers could be a possible solution. During the pre-operative phase of a difficult glenoid erosion, bone graft should be anticipated with the proper morphing, and checked during the procedure.

On the humeral side, retroversion and the contact point with the glenoid component should be studied for complete navigation in the future; the length and lateral off-set of the humeral component need to be properly planned, to avoid over-tensioning of the plexus, cuff and deltoid. Other drawbacks are the increasing operating times due to registration of anatomical landmarks, and increasing costs.

Some surgeons, especially experts and skilled ones, will probably consider CAS a waste of time and not necessary. However, similar concerns are usually raised when new technologies are introduced into surgical practice. This reluctance is usually overcome when the entire surgical team becomes confident and recognizes its potential.

### Can we guess future directions?

The lesson we learned from robotic hip and knee surgery is that a better intra-operative accuracy can be achieved with a less invasive surgery, avoiding cutting jigs and drilling guides, thanks to haptic control. The real advantage of robotics is the haptic technology that permits fine control of a small burr or reamer mounted on a robotic arm, through a smaller incision, without violating the safe volume [7].

Further steps will be developed for planning the humeral side and adapting soft tissue tension. An ideal pre-operative functional evaluation of patients could be associated with imaging: kinematics of the scapula and gleno-humeral joint can be assessed by sensors, and pre-operative planning might merge kinematic data with bone morphology. This would result in a patient-specific implant, with a functionally correct relationship between the glenoid and humeral components.

Alongside the pre-operative and intra-operative assessment, development technologies will also need to involve the post-operative follow-up of patients. The replaced joint’s kinematics can be monitored over time and compared with the initial functional evaluations and pre-operative planning images, in order to recognize possible early failures of the implant.

We are only at the beginning of a new and exciting era.

## References

[1] Mansat P, Barea C, Hobatho MC, Darmana R, Mansat M (1998). Anatomic variation of the mechanical properties of the glenoid. J Shoulder Elbow Surg.

[2] Walch G, Badet R, Boulahia A, Khoury A (1999). Morphologic study of the glenoid in primary glenohumeral osteoarthritis. J Arthroplasty.

[3] Villatte G, Muller A, Pereira B, Reilly P, Emery R (2018). Use of patient-specific instrumentation (PSI) for glenoid component positioning in shoulder arthroplasty. A systematic review and meta-analysis. PLoS ONE.

[4] Iannotti J, Baker J, Rodriguez E (2014). Three-dimensional preoperative planning software and a novel information transfer technology improve glenoid component positioning. J Bone Joint Surg Am.

[5] Kircher J, Wiedemann M, Magosch P, Lichtenberg S, Habermeyer P (2009). Improved accuracy of glenoid positioning in total shoulder arthroplasty with intraoperative navigation: a prospective-randomized clinical study. J Shoulder Elbow Surg.

[6] Verborgt O, De Smedt T, Vanhees M, Clockaerts S, Parizel PM, Van Glabbeek F (2011). Accuracy of placement of the glenoid component in reversed shoulder arthroplasty with and without navigation. J Shoulder Elbow Surg.

[7] Lang JE, Mannava S, Floyd AJ (2011). Robotic systems in orthopaedic surgery. J Bone Joint Surg Br.

